# Dynamics of food sources, ecotypic distribution and *Trypanosoma cruzi* infection in *Triatoma brasiliensis* from the northeast of Brazil

**DOI:** 10.1371/journal.pntd.0008735

**Published:** 2020-09-28

**Authors:** Maurício Lilioso, Carolina Reigada, Dayane Pires-Silva, Fernanda von H. M. Fontes, Cleanne Limeira, Jackeline Monsalve-Lara, Elaine Folly-Ramos, Myriam Harry, Jane Costa, Carlos Eduardo Almeida

**Affiliations:** 1 Instituto de Biologia, Universidade Estadual de Campinas – UNICAMP, Brazil; 2 Centro de Ciências Biológicas e da Saúde, Departamento de Ecologia e Biologia Evolutiva, Universidade Federal de São Carlos – UFSCAR, Brazil; 3 Universidade Federal da Paraíba, Campus IV, Brazil; 4 Université Paris-Saclay, CNRS, IRD, UMR Évolution, Génomes, Comportement et Écologie, Gif-sur-Yvette, France; 5 Laboratório de Biodiversidade Entomológica, Instituto Oswaldo Cruz, Fiocruz, Av. Brasil, Manguinhos, Rio de Janeiro, Brazil; Fundacao Oswaldo Cruz Instituto Rene Rachou, BRAZIL

## Abstract

Innovative approaches used to combat Chagas disease transmission tend to combine a set of comprehensive efforts to understand the ecology of local vectors. In this work we identified molecularly the blood meal of 181 *Triatoma brasiliensis*, distributed in 18 populations (8 sylvatic and 10 peridomestic), which were collected across a range of 240 km (East-West) and 95 km (North-South) in the semi-arid region of northeastern, Brazil. We used the vertebrate mitochondrial gene (cytochrome B) sequencing applied to DNA isolated from bug midgut to identify the insect blood meal sources via the BLAST procedure. The peridomestic populations were classified according to two main hypotheses of site-occupancy for *T*. *brasiliensis*: the first says that the infestation is mainly driven by structures that resemble its natural habitat (stony-like ecotopes) and the second assumes that it is associated with key-hosts (rodents and goats). Rodents of the Caviidae family (*Galea spixii* and *Kerodon rupestris*) were identified as the key-host of *T*. *brasiliensis*, but also the potential *Trypanosoma cruzi* reservoir–able to connect the sylvatic and domestic *T*. *cruzi* cycle. Cats also deserve to be studied better, as potential *T*. *cruzi* reservoirs. By modeling the food sources + site-occupancy + *T*. *cruzi* natural infection, we identified man-made ecotopes suitable for forming dense triatomine infestations with high rates of *T*. *cruzi* natural infection, which may be taken into account for vector control measures.

## Author summary

Blood-sucking bugs are vectors of the parasite *Trypanosoma cruzi*, which causes the Chagas disease. *Triatoma brasiliensis* is the main Chagas disease vector in the Caatinga eco-region of northeastern Brazil. Previous studies have already indicated that this species is associated with rodents of the Caviidae family–mainly in the sylvatic environment. In this study, we showed that these rodents are the main food sources of *T*. *brasiliensis* also in peridomestic environments. This association is frequently followed by numerous populations of *T*. *cruzi* infected insects in man-made ecotopes to store materials of mineral and wood origin. We suggest that the key-host that drives these infestations are mainly *Galea spixii*, followed by *Kerodon rupestris* (both Rodentia: Caviidae), which seem to be in synanthropic processes because they were found as frequent blood meals for *T*. *brasiliensis* in the sylvatic and domestic environments. Other mammals–mainly cats–may play an important role in Chagas disease eco-epidemiology for also being an important source of food for triatomines in the sylvatic and peridomestic environments. We conclude that educational programs to avoid Chagas disease transmission should include advising local people to avoid storing wood and tiles/bricks in the peridomestic environment–shelters for key-host rodents. Besides, they must be aware that these cavies may be infected by *T*. *cruzi*; and cautions are needed if they are to be used as a source of protein by hunting.

## Introduction

In Latina America, about eight million people are infected by Chagas disease and approximately 25 million are at risk, placing this endemic disease among the most serious parasitic illnesses in the Southern Hemisphere. Chagas disease is considered a neglected disease, which results in a chronic condition with high morbidity and mortality. It results in a high negative social and economic impact [1]. The disease is classically transmitted through blood-sucking bugs (Hemiptera: Reduviidae: Triatominae) infected with the protozoan parasite *Trypanosoma cruzi*—the causative agent of Chagas disease. Vector control measures have decreased the classic Chagas disease transmission in the whole of Latin America thanks to international efforts from Southern Cone, Central America, Andean Pact and Amazonian Intergovernmental Initiatives [1].

Whereas classic Chagas disease transmission is decreasing, oral contamination is taking place, even in areas previously considered non-endemic [2]. It is important to highlight that oral transmission is also vector dependent–as infected insects contaminate food and drinks. In Brazil, *Triatoma brasiliensis* is the most important Chagas disease vector in semi-arid areas of the northeast because it keeps sylvatic foci in rocky outcrops and is also adapted to infest human dwellings [3]. Additionally, infected populations infest several municipalities from five states of that region [3,4]. This species remains an operational challenge for vector control because it continually invades and colonizes human domiciles [5,6]. This triatomine was probably involved in recent Chagas disease outbreaks [7] as it was found with high densities and *T*. *cruzi* prevalence in domestic and peridomestic habitats around the outbreak area [8]. The difficulty to control *T*. *brasiliensis* is attributed to its capacity to occupy the domestic, peridomestic and sylvatic environment [9]. Gene flow between sylvatic and peridomestic/domestic populations, with a high prevalence of *T*. *cruzi* infection has been shown by mitochondrial gene and microsatellite variations [5]. This poses a threat for vector control efforts because sylvatic populations represent perennial foci.

Triatomines with the capacity to colonize man-made environments are considered ‘synanthropic’ [6,10]. For *T*. *brasiliensis*–a primary inhabitant of rocky-outcrops–the force exerted by the composition of peri-household micro-habitat [11,12] is proposed, as it is believed that these insects have adapted to peridomestic and domestic habitats because these environments contain rock-like (mineral) man-made structures that resemble its natural habitat. This hypothesis is hereafter referred to as “micro-habitat” hypothesis. However, the association with key-hosts has also been proposed, mentioned here as the “key-host” hypothesis. Some authors [13,14] found stronger support with the view that *T*. *brasiliensis* colonization of anthropic sites is driven by key-hosts (mainly rodents and goats), but the inferences made on the key hosts were thus far based only on field observations.

In light of the limitations of the above-mentioned approach; precise food source recognition of triatomines can provide crucial information to understand the eco-epidemiology of Chagas disease, by evaluating the risk of human infection and guiding the development of more effective vector control strategies [5]. These methods can provide a great contribution to test the hypotheses of site-occupancy, as the food source preferences can be objectively identified. Controversially, the blood meal detection/inferences to test the site-occupancy hypotheses for *T*. *brasiliensis* has been applied only locally (*i*.*e*. in small geographic scales) [5,14,15,16] and/or are based only on site-observation [14]. Here, the molecular identification of food sources was applied in a wider geographic area, which was implemented with field information on the site-occupancy of *T*. *brasiliensis* and molecular *T*. *cruzi* detection to infer on the potential reservoirs in ecotopes, in addition to vector (*T*. *brasiliensis*) and parasite (*T*. *cruzi*) distribution patterns.

## Materials and methods

### Insects

**Fig 1:**
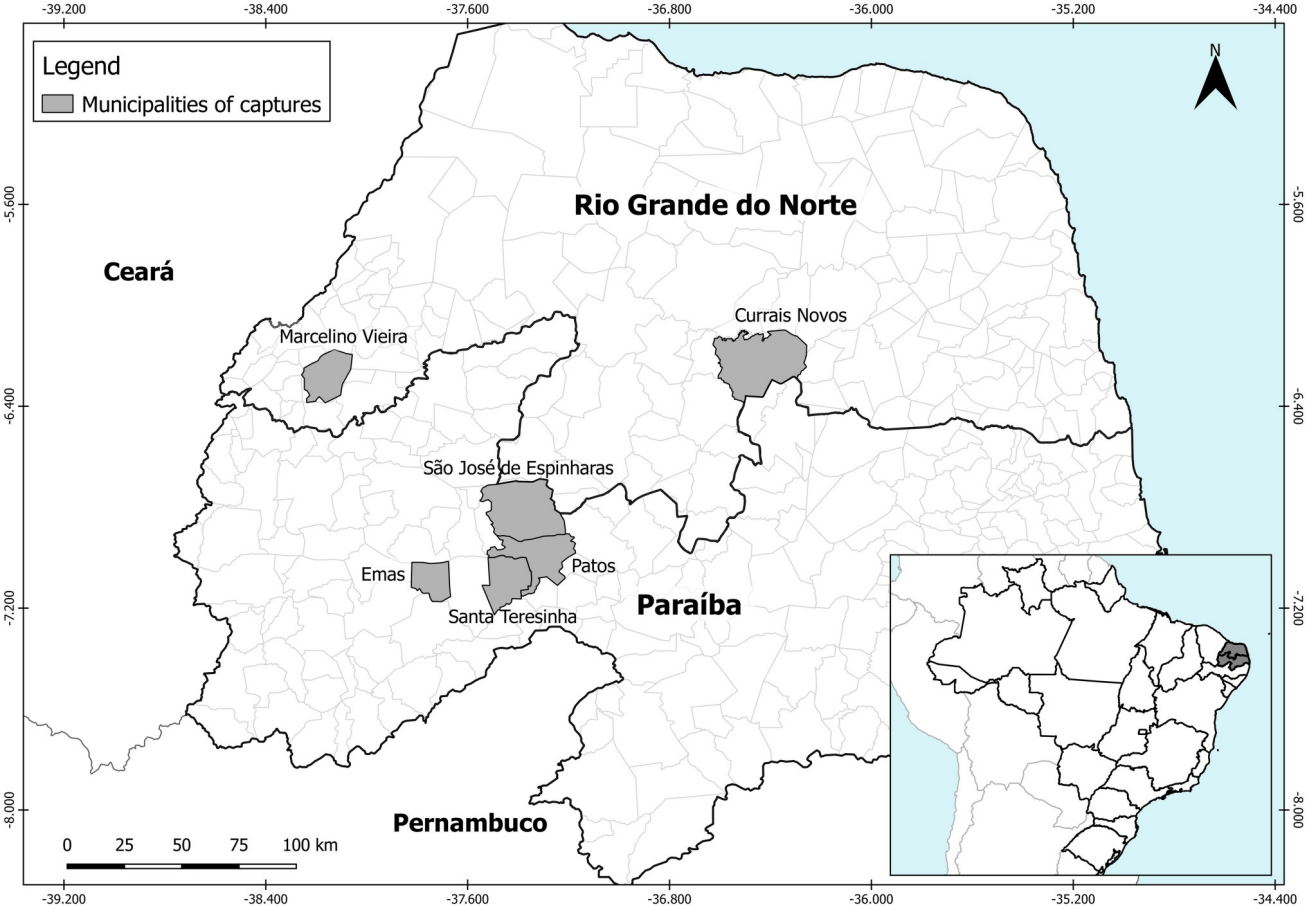
In gray, it is shown the States and Municipalities where *Triatoma brasiliensis* samples were collected in the states of Rio Grande do Norte and Paraíba, Brazil.

Triatomine captures were conducted in the states of Rio Grande do Norte (RN) and Paraíba (PB), Brazil, in the dry period (December to February). In RN, they were conducted in the municipalities of Currais Novos and Marcelino Vieira. In PB, they occurred in the municipalities of Emas, Patos, Santa Teresinha and São José de Espinharas. The collection area comprised a range of 240 km (East-West) and 95 km (North-South) (-06°58'48',0" to -06°08'49,2" latitude and -38°35'27,6"to-36°29'09,6" longitude) (Fig 1). All sampled spots were within the bio-geographic zone known as Caatinga–a mosaic of xerophytic, deciduous, semiarid thorn scrub, and forest [17]. The peridomestic ecotopes were defined as the spaces surrounding domiciles, where domesticated animals sleep or are raised. In peridomiciles, most of the triatomines were captured in stone/woodpiles, chicken coops, goat and pig corrals. Sylvatic ecotopes were represented by rocky outcrops, although we investigated for *T*. *brasiliensis* in other kinds of ecotopes, such as cacti, bird nests, and palms where no specimens were found. Triatomine capture was performed by exhaustion: all bugs seen were caught with the aid of tweezers/gloves by three researchers and one technician. Peridomestic and sylvatic populations were sampled in each municipality; the peridomestic captures were performed along the day, whereas sylvatic searches were nocturnal during the same field expeditions. During these captures, we took note of observed vertebrates and vestiges (feces), as a source for potential food sources. Collected specimens were identified according to taxonomic keys [18,19].

We asked residents about the use of sylvatic animals for hunting and as pets. We obtained permission from house owners/residents to search for insects on their properties and visited four houses in each spot (permission “Informed Consent Form” “TCLE 2.665.746”). The duration of each observation was about an hour and a half in each house (varying ±15 min). With the same team, we performed captures in sylvatic environments, where triatomines could be found in rocky outcrops–starting at the sunset (∼5 PM). The task took approximately five hours (varying ±30 min) in each spot. Insects were stored in 100% alcohol and frozen in the field to avoid blood digestion.

### Assumptions to select the sampling

We discarded the starved insects and populations with less than 30 samples. We also privileged nymphs in the last stages (N4-N5; 84% of the total selected), but we had to take some youngers (10%). Therefore, immature insects comprised 94% of total sampling. This procedure was conducted because they are expected to be more sessile, as non-flyer forms and we aimed to privilege resident insects, instead of invaders. This strategy aimed to decrease sampling bias regarding the associations between the food source and the ecotopes. The criterium to select non-starving insects was based on the visualization of the blood meal (a black mass with more than 20–25 mg) during dissection, assuming the well-fed insects have more chances to be residents. We replaced insects which we could not have the information on blood meals (e.g. unsuccessful PCRs/sequencing or mixed feedings) by untested samples from the same population. This procedure aimed to keep the proportion (10–11%) of samples molecularly analyzed to perform inferences on the population size.

### Population definition

Samples collected in the same spot and ecotope were defined as populations, identified by their acronyms: (i) the first two letters were referring to the cities: Currais Novos-RN (CN), Marcelino Vieira-RN (MV), Emas-PB (EM), Patos-PB (PT), Santa Teresinha-PB (ST) and São José de Espinharas-PB (SJ) (Fig 1); (ii) the numbers referred to the geographical coordinate; (iii) the last letter defined the ecotope: sylvatic (S) and peridomestic (P) (S1 File).

### Molecular blood meal identification

The insects were dissected to remove the abdominal contents, which were frozen in liquid nitrogen to be individually mashed with a sterile crusher. We used the Dneasy Blood and Tissue Kit (Qiagen) for DNA extraction, following the manufacturer´s recommended protocol. We sequenced a fragment of mitochondrial cytochrome b (cyt b) gene using vertebrate-specific primers L14841 / H15149, following the cycles of amplification recommended [20]. We performed the PCRs, but the PCR purification process, direct sequencing, as well as sequencing reactions, were performed by the company Macrogen (Seoul, Korea). Both strands were sequenced, which were then assembled into contigs for edition [21]. All sequences had more than 200 pb (mean 354 pb), which were submitted to BLAST (https://blast.ncbi.nlm.nih.gov/Blast.cgi). We used sequences with values of > 95% identity and 10^−100^ E -value.

### Natural infection by *Trypanosoma cruzi*

To identify the natural infection by *T*. *cruzi*, the same DNA from the insects' abdominal contents mentioned in previous steps were used with the primers TcH2AF and TcH2AR [22], present in the 3' non-encoding region of the 1.2 kb unit encoding for histone H2A from *T*. *cruzi*. The PCR reactions were performed in a 25 μL final volume, with 5x Green GoTaq Buffer, 1.5 mM of MgCl_2_, 1U of GoTaq DNA Polymerase, 10 pmol of each primer, 200 μM of dNTP and 25–50 ng of DNA. For amplification, PCRs were performed within 29 cycles (95°C/ 5 min, 95°C/ 1 min, 61°C/ 30 s and 72°C/ 1 min) and a final incubation of 72°C for five minutes. The PCR products were applied to a 2% agarose gel and visualized with ethidium bromide staining. The samples that showed a fragment (band) with 234 bp were selected as positive for the presence of *T*. *cruzi*. In all set of PCRs we included a DNA extracted from a *T*. *cruzi* (TcI) culture as a positive control. Additionally, the negative controls used were NTC (non-template control) and a *T*. *rangeli* DNA identified and isolated from *T*. *brasiliensis* [23]. We assumed that these DNAs did not have inhibitors for PCR amplification as we used the same insects’ DNAs that were successfully amplified for feeding source detections (see the previous topic).

### Food source composition and diversity

To assess blood meal sources’ diversity, Shannonʼs diversity index $H^{'}=-\sum_{i=1}^{S}\left(\frac{n_{i}}{N}\right)\;\text{ln}\;\;\left(\frac{n_{i}}{N}\right)$ was calculated, with “ni” representing the number of individuals of species/taxon “I”, “N” the total number of individuals, and “S” the total number of species/taxa [24]. For evaluating the richness of vertebrate species used as blood sources by *T*. *brasiliensis* in the different ecotopes, rarefaction curves were elaborated upon using the software Past v.3.23 and Microsoft Excel. The diversity of blood meals found was compared using a Student’s t-test, considering that the test result was significant if P < 0.05 [25]. The blood meal composition among sites and ecotopes was also evaluated by chi-square tests or Fisher’s exact tests, considering that the test results were significant when P < 0.05.

### Food sources at the ecotypic level and *Trypanosoma cruzi* infection prevalence

To explore trophic sources, the ecotypic distribution of blood meal and the vector interaction with *T*. *cruzi* regarding each blood-feeding; a network was constructed using Cytoscape 3.7.2 [26], where the blood meal and the insect populations were represented by nodes.

### Ecotypic site-occupancy

**Fig 2:**
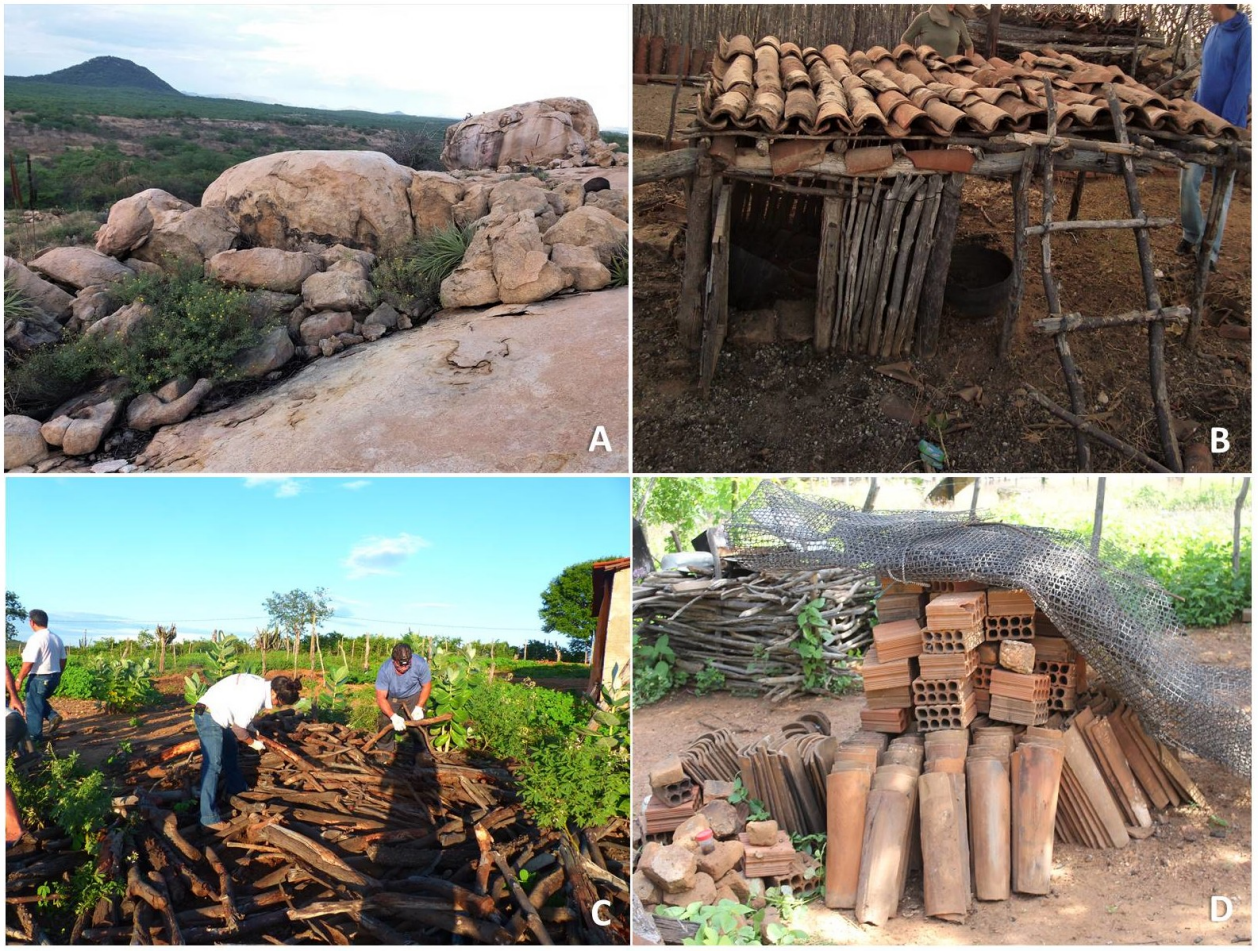
Ecotopes where *Triatoma brasiliensis* is found. A) natural habitat (the primary ecotope), represented by rocky outcrops. The artificial habitats are: B) breeding places for livestock; C) woodpiles, used for wood-fired ovens; and D) piles of the material of mineral origin (tiles, bricks and stones), used mainly for building constructions.

To test the hypothesis that *T*. *brasiliensis* exhibits a site-occupancy preference for stone-like materials, we built a simpler ecotypic characterization than the one presented by authors [14]. This modification was based on the assumption that man-made ecotopes for livestock are occupied by predictable hosts–mainly of exotic Brazilian fauna (e.g. chicken, cows, dogs, goats and sheep). Therefore, the first ecotypic group was characterized by the natural (primary) ecotope of species in the *T*. *brasiliensis* complex–rocky outcrops (Fig 2A). Artificial (secondary) ecotopes are the man-made structures, which were defined as (i) breeding sites for livestock (mainly chicken, goats, sheep, dogs and cows–frequently built of mixed material: mineral and woody) (Fig 2B), (ii) woodpiles (Fig 2C)–which are mainly used to supply wood-fired ovens and (iii) piles of the material of mineral origin (tiles, bricks and stones) (Fig 2D), which are accumulated for the construction of houses, roofs, walls and roads.

### Statistical analyses

The frequency of occurrence of *T*. *brasiliensis* and the infection rates of insects by *T*. *cruz*i were analyzed by adopting generalized linear models (GLM) with Poisson and Binomial error distribution corrected for overdispersion, respectively, including the effects of habitat, food source, *T*. *cruzi* natural infection and locality of collection. The significance of the habitat, blood meal and collection location was assessed through analyses of deviance tables. Multiple comparisons were performed by obtaining 95% confidence intervals for linear predictors. Goodness-of-fit was assessed using half-normal plots with simulation envelops [27]. Principal component analysis (PCA) was conducted to rank the three groups of animals according to their importance as the source of food for *T*. *brasiliensis* and consequently, their potential as *T*. *cruzi* reservoirs. Previous studies showed that rodents of Caviidae family are the main food sources of *T*. *brasiliensis* in the sylvatic habitat [5,14,15,18], sometimes found in peridomestic environments [14]. We combined all other mammals because they are also potential *T*. *cruzi* reservoirs, but are mainly exotics of Brazilian fauna (as cats, dogs, goats and cows). The last group was composed of refractory animals to *T*. *cruzi* (birds and reptiles). For all analyses, the statistical program R (https://www.r-project.org/) was used.

## Results

### Blood meal detection

A total of 3,897 triatomines were captured. Selected insects were distributed among 18 populations. This selection followed our assumptions (see Material and methods). Therefore, we identified the blood meal of 181 *T*. *brasiliensis*, of which the majority was N5 (59%), followed by N4 (25%), N3 (9%), adult males (4%), adult females (2%) and N2 (1%) (S1 File). Selected insects belonged to eight sylvatic populations and ten peridomestic. Of these, 70 (39%) are from triatomines captured in the wild and 111 (61%) in domiciliary unities (peridomestic ecotopes). Gene fragment amplifications worked for 85% of the samples selected at the first time we tested. The remaining 15% were replaced by untested specimens from the same populations until our assumptions were achieved. One sample sequenced *T*. *brasiliensis* and two mixed feeds were observed–characterized by double nucleotide peaks (ambiguous) in the chromatograms in a large proportion of the DNA fragment–which were also replaced. Nineteen species of blood meal sources of *T*. *brasiliensis* were recognized, distributed in 15 families, of which 127 (70%) were rodents of Caviidae family: *Galea spixii* (64%, 116/181), here refereed as “common cavy”, but also known as “Spix's yellow-toothed cavy”; and *Kerodon rupestris* (6%, 11/181), here referred to as “rocky cavy” and both are referred to as cavies. Cats also had a high representation (6%, 11/181). The proportion of blood meals was also observed at an ecotypic level. Cavies accounted for more than 62% of blood meals in both environments (Table 1, S1 and S2 Files). Even small sylvatic populations had fed on more than one food source. On the contrary, some large peridomestic populations had fed on a single food source–or a limited number of food sources. It was confirmed via Shannonʼs diversity index: it was 0.47 for the peridomestic environment whereas for the sylvatic it was 0.79 (*d*.*f*. = 1, p<0.005).

**Table 1:** Identification of the *Triatoma brasiliensis* food source of populations captured in the states of Rio Grande do Norte and Paraíba, Brazil. Individual information from each insect (*i*.*e*. insect population, ecotope, municipality, state, positivity for *T*. *cruzi* infection and BLAST identity, among others) is detailed in S1 File.

Food sources					Ecotopes		
Class	Family	Species	Common name	NI (%)	Syl (N)	Peri (N)	Total
Mammalia	Caviidae	*Galea spixii*	Common cavy	57	36	80	116
		*Kerodon rupestris*	Rocky cavy	91	7	4	11
	Felidae	*Felis catus*	Cat	91	6	5	11
	Didelphidae	*Didelphis albiventris*	Opossum	100	0	4	4
	Suidae	*Sus scrofa*	Pig	75	0	4	4
	Muridae	*Mus musculus*	Mouse	0	2	1	3
	Echimyidae	*Thrichomys apereoides*	Sylvatic rodents	25	3	0	3
		*Thrichomys inermis*			1	0	1
	Bovidae	*Bos taurus*	Cow	100	1	0	1
	Procyonidae	*Procyon cancrivorus*	Raccoon	100	1	0	1
Reptilia	Tropiduridae	*Tropidurus semitaeniatus*	Lizards	64	3	0	3
		*Tropidurus hispidus*			1	1	2
	Teiidae	*Tupinambis merianae*			5	0	5
	Phyllodactylidae	*Phyllopezus periosus*			1	0	1
	Dipsadidae	*Philodryas nattereri*	Snake	0	0	1	1
Birds	Phasianidae	*Gallus gallus*	Chicken	27	0	11	11
	Thraupidae	*Dubusia* sp.*	Sylvatic bird	100	1	0	1
	Columbidae	*Columbina picui*	Sylvatic doves	0	1	0	1
		*Zenaida auriculata*			1	0	1
				**Total**	70	111	**181**

Syl = sylvatic; Peri = peridomestic; N = number of food sources detected; NI = natural infection prevalence by *T*. *cruzi* for each insect with food source detected.

*uncertain identification

### *Trypanosoma cruzi* natural infection

Parasite infection was counted only for the insects from which the blood meals could be detected. Of the 181 insects, 59% were infected by *T*. *cruzi*; of this 40% (73/181) originated from peridomestic environments and 19% (34/181) came from sylvatic environments. (S1 File).

### *Trypanosoma cruzi* natural infection vs blood meals

**Fig 3:**
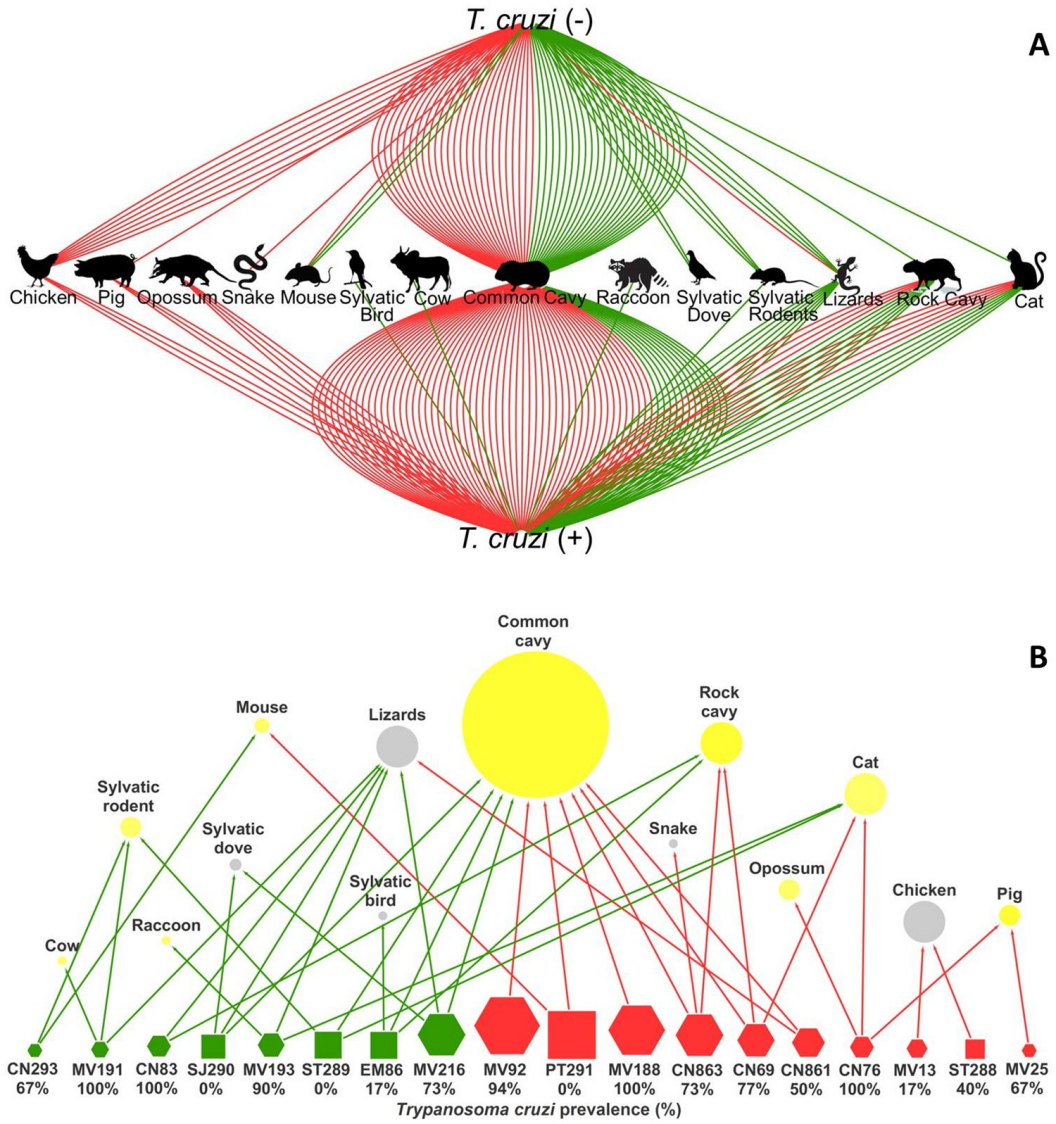
Overall food sources found for the 181 *Triatoma brasiliensis*. Connections (A) to the above pole represent food sources in insects infected by *Trypanosoma cruzi* and to the up pole non-infected insects. (B) illustrates the diversity of food sources per population, where *T*. *cruzi* refractory animals are in grey and potential reservoirs (mammals) are in yellow circles. Squares represent *T*. *brasiliensis* populations from Paraíba and hexagons the ones from Rio Grande do Norte State. For A and B the feeding sources found in the peridomestic population are in red lines/nodes and the sylvatic in green. The node sizes are proportional to the sampling size.

Each line in Fig 3A represents a blood meal detection, which is connected to an insect *T*. *cruzi* positive or negative. As mentioned above, considering the diversity of blood meals per insect population, the common cavy was the most frequent food source, represented in 56% (10/18) of populations (six peridomestic and four sylvatic), whereas cats and rocky cavies served as food for four populations (22%; 4/18; two peridomestic and two sylvatic). The common cavy was the unique food source for two peridomestic populations (MV92P and MV188P) which were highly infected by *T*. *cruzi* (94–100%). Two other peridomestic population (CN69P and CN863P) fed mainly on the common cavy and the rocky cavy, with a few feedings on cats and on a snake–the latter is *T*. *cruzi* refractory. However, both exhibited 73–77% of *T*. *cruzi* prevalence. Cats were detected as blood meals with the same proportion that rocky cavies–both usually found in highly infected bug populations in peridomestic and sylvatic environments (S1 and S2 Files). Reptiles served as food sources for six *T*. *brasiliensis* populations, of which only one was peridomestic. These animals are refractory to *T*. *cruzi* infection, but 80% (8/10) of bugs that fed on these lizards were infected. In the network (Fig 3B) it is possible to observe that all bug populations that fed on lizards had insect that fed also in mammals, indicating that lizards may be an alternative source of food–explaining also the *T*. *cruzi* positivity. In the same way, chicken–also refractory to *T*. *cruzi* infection–served as a food source for two bug populations, comprising 6% (11/181) of insects. Of these, three were infected, indicating that those triatomines must have fed on a *T*. *cruzi* reservoir before feeding on chicken. Indeed, rodents (probably *Thrichomys* sp.) were observed in chicken crops while capturing insects.

### Feeding source vs Natural infection per state

In Rio Grande do Norte state, 131 insects were collected, of which 103 (79%) were infected. The profile was different for the Paraiba state. Only four of 50 (8%) insects collected were infected. Bugs that fed on cavies from Rio Grande do Norte always had more than 73% of natural infection. One of the sylvatic insect populations from Paraíba had 11% (2/12) of natural infection. In this population, almost all bugs (11) fed on cavies (common cavy N = 10; rocky cavy N = 1)–the only exception was one insect that fed on a sylvatic pigeon (*Columbina picu*i). However, none of the 14 insects that fed on the common cavy in a peridomestic population from Paraíba (PT291P) were infected by *T*. *cruzi* (Fig 3B). The ten analyzed *T*. *brasiliensis* of other populations from Paraíba (EM86S) fed on the common cavy and had 20% (2/10) infected bugs. Despite the disparity in the prevalence of *T*. *cruzi* infection between the states of Rio Grande do Norte (79%, 103/131) and Paraíba (8%. 4/50), our data (Fig 3) did not differ in the composition of blood meals between bug populations from both states, which was confirmed via Chi-squared tests (X^2^ = 2.96, *d*.*f*. = 1, P = 0.22).

### *Triatoma brasiliensis* frequency of occurrence

**Fig 4:**
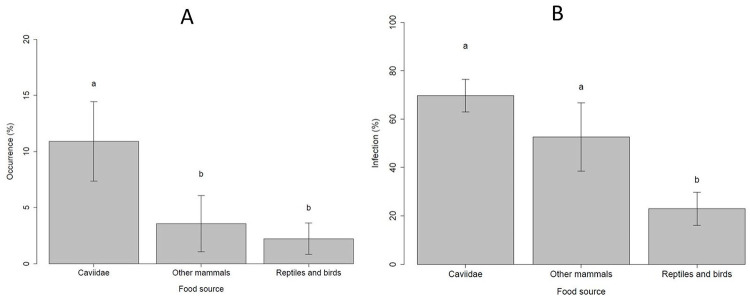
(A) Frequency of occurrence (%) of *Triatoma brasiliensis* on different food sources. (B) Infection rates (%) of *Triatoma brasiliensis* by *Trypanosoma cruzi* fed on the different food sources analyzed in several sites of Rio Grande do Norte (RN) and Paraíba (PB) states, Brazil. Distinct letters point to statistical differences (p<0.05, Tukey).

According to the epidemiologic importance and frequency of blood meals found for *T*. *brasiliensis*, we consider three main groups of blood meals: (i) cavies (predominant blood meal), (ii) other mammals (potential reservoirs) and (iii) reptiles and birds (*T*. *cruzi* refractory). The occurrence of *T*. *brasiliensis* was influenced by the type of source of food present at sample sites (F_23,25_ = 28.197, p<0.005). Cavies were the most abundant source of food for *T*. *brasiliensis*, compared to others (Fig 4A). The effects of locality and habitat were not significant on the occurrence of *T*. *brasiliensis* (F_5,20_ = 1.43, p = 0.2673; F_20,23_ = 1.8237, p = 0.1753, respectively).

### Infection rates of *Triatoma brasiliensis* by *Trypanosoma cruzi*

The effects of locality and habitat were not significant on infection rates of insects by *T*. *cruzi* (F_10,13_ = 1.73, p = 0.22; F_13,16_ = 1.01, p = 0.42, respectively). The food source affected the *T*. *cruzi* infection prevalence of *T*. *brasiliensis* (F_16,18_ = 4.78, p<0.05). The infection prevalence in insects fed on cavies and other mammals were higher than those insects fed on reptiles and birds, as expected (4B).

**Fig 5:**
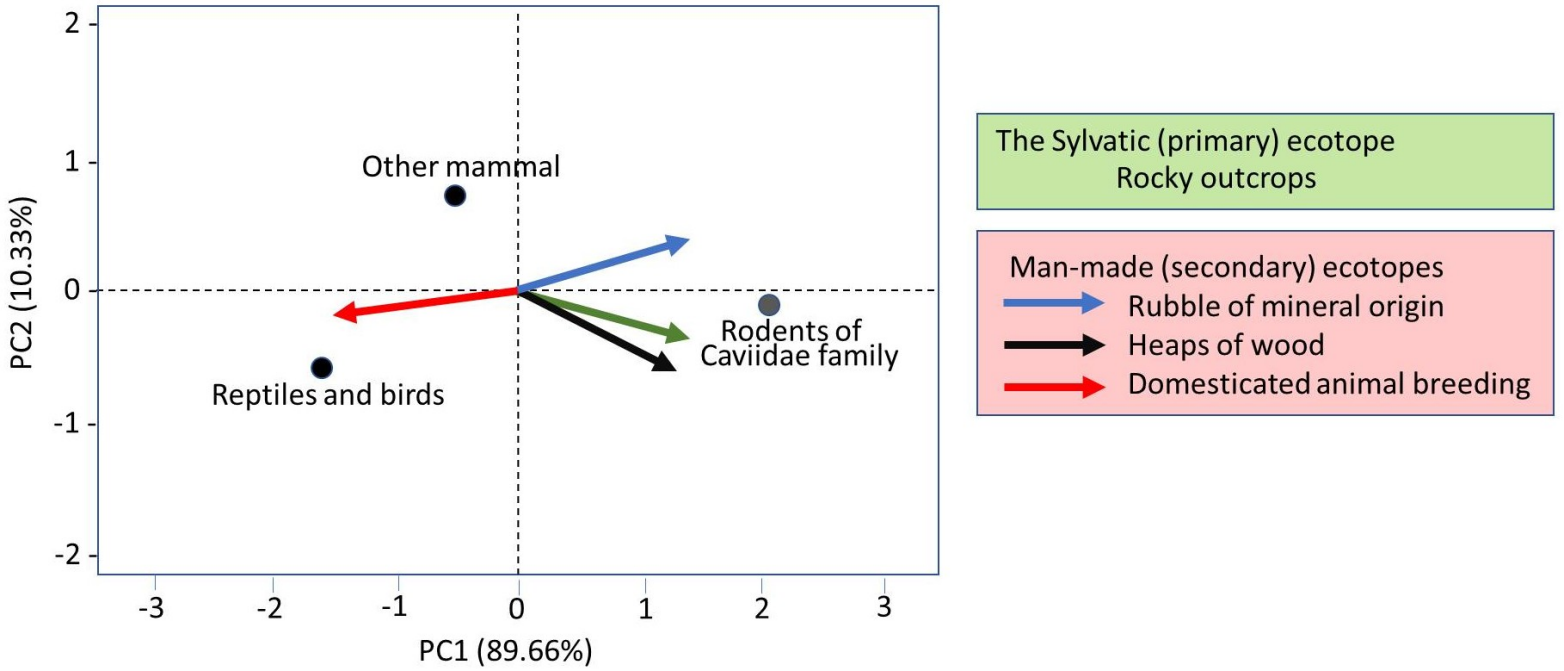
The main *Triatoma brasiliensis* food sources detected in each group of ecotope: The natural (rocky outcrops) and the artificial (mineral piles, woodpiles and livestock breeding) colonized by *T*. *brasiliensis* in the Rio Grande do Norte (RN) and Paraíba (PB) states analyzed by principal component analysis.

The PCA was conducted to evaluate the association of three groups of animals detected molecularly as sources of food for *T*. *brasiliensis* and habitats of these insects. The PCA results (Fig 5) indicate that *T*. *brasiliensis* that fed on cavies were almost so associated with man-made ecotopes as they were with their natural/primary habitats (rocky-outcrops). Additionally, cavies did not exhibit evident choice for any of the two man-made ecotopes to store material (tile piles or woodpiles). On the other hand, insects that fed on livestock and reptiles appeared in the opposite pole of the x-axis of PC1.

## Discussion

The molecular detection of blood meals for *T*. *brasiliensis* was already explored, but these studies were limited to small geographic scales [5,15,28]. A detailed study on site-occupancy on *T*. *brasiliensis* has already been presented with robust findings, but with subjective inferences on the food sources [14]. The power to determine the vectors available to connect the sylvatic and domestic transmission cycles may be improved if the modeling is combined with an objective way to determine the blood meal–by using molecular approaches [5]. Additionally, we combined this information with *T*. *cruzi* prevalence–which we demonstrated to be significantly distinct, according to the states; and with site-occupancy in the peridomestic environment. Therefore, we hereby indicate some “sylvatic” rodents of Caviidae family as potential *T*. *cruzi* reservoirs, also in the peridomestic environment. Cats should also be better investigated as potential *T*. *cruzi* reservoirs. Additionally, we presented information with the potential to be taken into account as part of integrated control strategies.

Nineteen species of blood meals of *T*. *brasiliensis* were recognized and distributed among 15 host families. Unexpected blood meals were found, such as snakes, raccoons, sylvatic birds and doves, indicating the great variety of *T*. *brasiliensis* feeding habits. Of the total blood meals detected, 127 (70%) belong to rodents of Caviidae family–a kind of food source that was already found in other studies [5,15]. As expected, the higher diversity of blood meals in the sylvatic environment was evidenced by Shannonʼs diversity index.

We found at least two large populations with high *T*. *cruzi* prevalence (>94%) that fed exclusively in cavies. One of these was the population MV188P with 100% of natural infection–collected in a woodpile aside (~50m) the sugar cane mill probably involved in the Chagas disease outbreak that was recently reported [7]. Additionally, *T*. *brasiliensis* populations from Rio Grande do Norte state associated with these rodents are usually also highly infected. We found a high prevalence of rodents considered sylvatic–mainly *G*. *spixii*, but also *K*. *rupestris*–as feeding sources for *T*. *brasiliensis* in a high proportion of peridomestic environments investigated (60%, 6/10). This indicates that in the studied areas they are getting closer to anthropogenic environments in a synanthropic process. Indeed, rodents of Caviidae family have been historically associated with the triatomine adaptation to anthropogenic habitats, as it is the case of *T*. *infestans*/*T*. *dimidiata* in the Andean valleys/Yucatan-Mexico by the domestication of guinea pigs (*Cavia* spp.) [29,30,31,32,33]. It is also well known that cavimorphs maintain high *T*. *cruzi* parasitemia [31,33,34,35]. In Caicó-RN, Brazil, it had already been highlighted the potential importance of rocky cavies (*K*. *rupestris*) in the *T*. *cruzi* cycle in the sylvatic environment [5], but we here found blood meals in rocky cavies in peridomestic *T*. *brasiliensis* populations. This indicates that these cavies are also circulating in both environments. In rural communities in Tafı´ del Valle–Tucuman Province, Argentina–peridomestic populations of *T*. *eratyrusiformis* were found infected by *T*. *cruzi* in dry-shrub fences associated with colonies of *Microcavia australis* (Rodentia:Caviidae) [32]. Some authors [33] suggest that this last cavy species is a highly competent host of *T*. *cruzi* I.

The diversity of blood meals found in this study was similar to the ones found for *T*. *dimidiata* in Yucatán, Mexico [29]. Indeed, as expected, they found a distinct set of mammals probably involved in *T*. *cruzi* exchanges between sylvatic and domestic environments, such as dogs, humans and cows. Overall, 28.7% of *T*. *dimidiata* were infected by *T*. *cruzi* [29]. A matter of concern is that we found a much higher prevalence of *T*. *cruzi* (59%) in *T*. *brasiliensis*, because a large proportion of *T*. *cruzi* infected bugs were physically closer (40%, 73/181) to humans–in peridomestic ecotopes. However, we did not detect any feeding on humans. This may be the result of a wide availability of food sources in the peridomestic environment, which may have been driven by our sampling strategy (see “Assumptions, hurdles and caveats”). Additionally, we did not detect blood meals on humans in the sylvatic environment. However, we did not see any nocturnal hunters during the fieldwork. During the day farmers may be circuating in the sylvatic environment. However, the rocks are too warm to allow the insects to be wondering over them, forcing insects to be hidden in protected sites within rocky outcrops [12].

Statistical analyses did not find a significant difference in the potential capacity of other mammals to keep *T*. *cruzi* if compared to cavids. It is not realistic to provide a campaign to local people to avoid livestock breeding because it is an essential source of livelihoods, such as income, employment, transport, agricultural traction, amongst others. We conclude that human activities that put the population at risk of contact with infected triatomines may be avoided via educational programs, as already pointed out by Zeledon and Vargas [36] in a scenario in Costa Rica. Additionally, the population must be aware of the potential risk of keeping sylvatic rodents as pets or for hunting–information provided by local people. For *T*. *cruzi* transmission by *T*. *dimidiata* in the Yucatan peninsula, Mexico, Flores-Ferrer and cols [37] developed sophisticated models of *T*. *cruzi* transmission in a multi-host combination. They suggested that vector reproduction and parasite transmission depend on the triatomine blood-feeding preference. Dogs were demonstrated to be key-hosts for *T*. *dimidiata* in Yucatan, concluding that if dog´s populations are reduced, the risk of Chagas incidence in humans can be significantly decreased too.

Cats appeared as food sources in four populations (2 sylvatic and 2 peridomestic) in both states (PB and RN). Only one bug that fed on cats was not *T*. *cruzi* infected. But as it can be seen in the networks, all *T*. *brasiliensis* that fed on cats fed also on opossum, rocky cavies, and common cavies, indicating that these bugs may have fed on other infected animals previously. In rural Northwestern Argentina, both dogs and cats showed a high and similar prevalence and incidence of *T*. *cruzi* infection and infectiousness for *T*. *infestans* [38]. These carnivores have higher chances of getting the infection by *T*. *cruzi* orally, such as by preying on infected rodents or by licking the fur with feaces of infected triatomines [39]. Cats have been neglected in serological surveys to look for sentinels of *T*. *cruzi* circulation. However, the role of cats in Chagas disease eco-epidemiology in Argentina has already been recognized [40]. Gürtler and Cardinal [40] reviewed the literature and found the occurrence of positivity for *T*. *cruzi* in parasitological tests for cats also in Venezuela [41] and Chile [42]. Interestingly, in this work, we did not find any feeding on dogs, which concurs with other studies [5, 15]. However, it has been considered a good sentinel of *T*. *cruzi* circulation in our study area; since some authors [43,44] have found that 40% of dogs were infected. This indicates that dogs may get infected by other means, such as orally. We recommend including cats as sentinels of *T*. *cruzi* circulation for future eco-epidemiological studies.

There is higher circulation of *T*. *cruzi* in Rio Grande do Norte state in comparison to other states which have already been suggested [3]. It was also shown that this high prevalence of natural infection is combined with high populational densities of *T*. *brasiliensis* in this state if compared to Paraíba [8]. These findings were confirmed by several authors throughout multiples approaches [23,43,44,45,46,47]. Noteworthy, even the populations that fed on cavies were not highly infected in Paraíba state. There is a need to understand what leads to the Paraíba state to exhibit lower *T*. *cruzi* circulation, even with insect feedings on potential parasite reservoirs.

Reptiles seem to be an alternative food source (6%, 11/181) for *T*. *brasiliensis* mainly in the sylvatic environment. Five sylvatic populations were found to feed on reptiles and one peridomestic. Recently, it was found that reptiles are the main food source (86%) for *T*. *petrocchiae* [45]*–*a triatomine that cohabits with *T*. *brasiliensis* [23]. Indeed, sporadic blood meals on cold-blooded animals (such as frogs) have already been observed for *T*. *brasiliensis* [5]. However, of the 86 sylvatic *T*. *brasiliensis* analyzed on a small geographic scale in the state of Ceará [15] only one bug fed on reptiles and none of the intradomestic or peridomestic had fed on this group of animals, reinforcing our finding that they represent an alternative source of food.

Experiments under lab-conditions used varied blood meals and provided important information on the *T*. *brasiliensis* bionomic advantages/disadvantages and attractiveness [48,49]. According to Flores-Ferrer and cols [50] these pieces of evidence can help to predict the potential for the triatomine adaptation to domestic environments. However, authors stressed [50] that results of lab-experiments must be interpreted with caution when extrapolating to natural situations. There are varied hypotheses to explain the domiciliation process (synanthropic process) in Triatomine and it is more likely that there is no single explanation for the adaptation of the entire diversity of species [51,52]–as well as multiple forces that may act in a combined manner. But for *T*. *brasiliensis* (a rupestrian species), the force exerted by the composition of the peri-household micro-habitat [12] is well accepted, as it is believed that these insects have adapted to the peridomestic habitats because these environments contain structures that resemble their natural habitats–in this case, of mineral construction. However, the association with key-hosts (synanthropic or domestic) has also been proposed [14, 18]. The ecological approach presented here aimed to test the role of both forces that assumed to drive adaptation/colonization to artificial ecotopes: the structure of the habitat and the food source. The PCA showed that bugs that had fed on cavies are almost so associated with human-made ecotopes to store stone-like materials and woods as they are to their natural habitat (rocky outcrops). In this sense, the key-hosts may play major roles in the site-occupancy for *T*. *brasiliensis*. However, this inference must be interpreted with caution because hosts are usually related to a variety of microhabitats. In any case, as *T*. *brasiliensis* populations associated with cavies exhibited a high prevalence of *T*. *cruzi* infection in both environments, there is evident potential for this association to exchange *T*. *cruzi* lineages between sylvatic and peridomestic ecotopes.

As the insect chemosensory system plays a key role in ecological adaptation to new or changing hosts or habitats, chemosensory genes represent candidates to understand the triatomine adaptation to anthropogenic systems [53–54]. For *T*. *brasiliensis*, the chemosensory genes, such as the odorant-binding proteins (OBPs), chemosensory proteins (CSPs), odorant receptors (ORs), or transient receptor potential channel (TRPs) were annotated in a reference transcriptome [53] and differential gene expression analysis demonstrated the under-expression in the chemosensory organs of the domiciliary bugs compared to the sylvatic ones [54]. Authors [54] associate this under-expression with the less diversity of xenobiotics and probably the more stable abiotic parameters in peridomiciliary environments than in sylvatic. However, considering the results here obtained, biotic parameters (as host odors) might play a major role in the synanthropic process of *T*. *brasiliensis* and deserve to be better investigated in future studies.

### Assumptions, hurdles and caveats

Regarding the assumption of selecting large and well-fed *T*. *brasiliensis* populations (N>30) we aimed to privilege well-installed colonies, which we assumed to be composed of more residents than invaders. Some studies [55] showed that in the absence of a host (e.g. host death, migration, removal) the insect population declines to the original refuge. Host-seeking dispersal starts happening one day after the host is removed in “semi-field” experimental conditions [55]. Additionally, in experimental conditions, it is shown that the blood meal might be undetectable after 45 days post-feeding [56]. We must consider that Caatinga is one of the Brazilian ecosystems with the highest temperature, especially in the dry period–which accelerates insect´s blood meal digestion. We assumed that this dispersion behavior may form populations with low nutritional statuses or with blood meals that might correspond to an ecotope, other than the ones in which the bugs were caught. Regarding the modeling of the site-occupancy hypothesis, the factors involved in the two competing hypotheses may not be treated so independently; and caution must be taken to interpret these results. We highlight that the connection of infected bugs with blood meals may not be used to assume mammals as reservoirs because insects might have been infected in other food sources before the blood meal was detected. Besides, our strategy of excluding populations with small sampling size (N<30) implies losing information on an important portion of the triatomine community with epidemiological importance in emerging foci. By selecting well-fed insects, we also think we avoided catching the ones with mixed feedings (observed for only two bugs–excluded and replaced by untested specimens)–which also have epidemiological meaning. Our sampling strategy did not allow surveying completely the dietary habits of *T*. *brasiliensis*, which should also include samples from the rainy season (April to August) and a larger number of blood meal identifications. However, the sampling size (N = 181 insects of 18 populations) was also limited if we take into account the number of variables that may be involved in the food sources of *T*. *brasiliensis* (e.g. evolutionary stages of insects, local fauna, etc.). Despite many of the above-listed limitations, we hereby showed how molecular epidemiology evidence may contribute to unravel situations potentially related to Chagasic transmission cycles by identifying associations among triatomine feeding sources, potential *T*. *cruzi* reservoirs and their ecotopes.

## Supporting information

S1 File *Triatoma brasiliensis* population, ID, municipality, state, evolutionary stage, ecotope, type of habitat, observed fauna in situ, *Trypanosoma cruzi Natural* infection, sequence length (bp), description BLASTED, animal BLASTED (common name), max score, total score, query cover, E-value, identity (%), accession, geographic coordinate, N [nymphal state], M = male and F = Female. (XLSX)

S2 File Blood meals found for the 181 *Triatoma brasiliensis* from 18 different populations collected in municipalities of Rio Grande do Norte (RN) and Paraíba (PB) states, Brazil. Overall blood meals (A) and divided by ecotope: Peri = peridomestic and Syl = sylvatic (B). Detailed information from each insect is in S1 File. Rodents of Caviidae Family are in bluish, other mammals are in greenish and reptiles with birds are in hot colors (orange to pinkish).
(TIF)
