# Supplementary figures and images for: Dynamics of food sources, ecotypic distribution and Trypanosoma cruzi infection in Triatoma brasiliensis from the northeast of Brazil
Source: PLoS Negl Trop Dis. 2020 Sep 28;14(9):e0008735. doi: 10.1371/journal.pntd.0008735 (PMC7575107; doi:10.1371/journal.pntd.0008735)

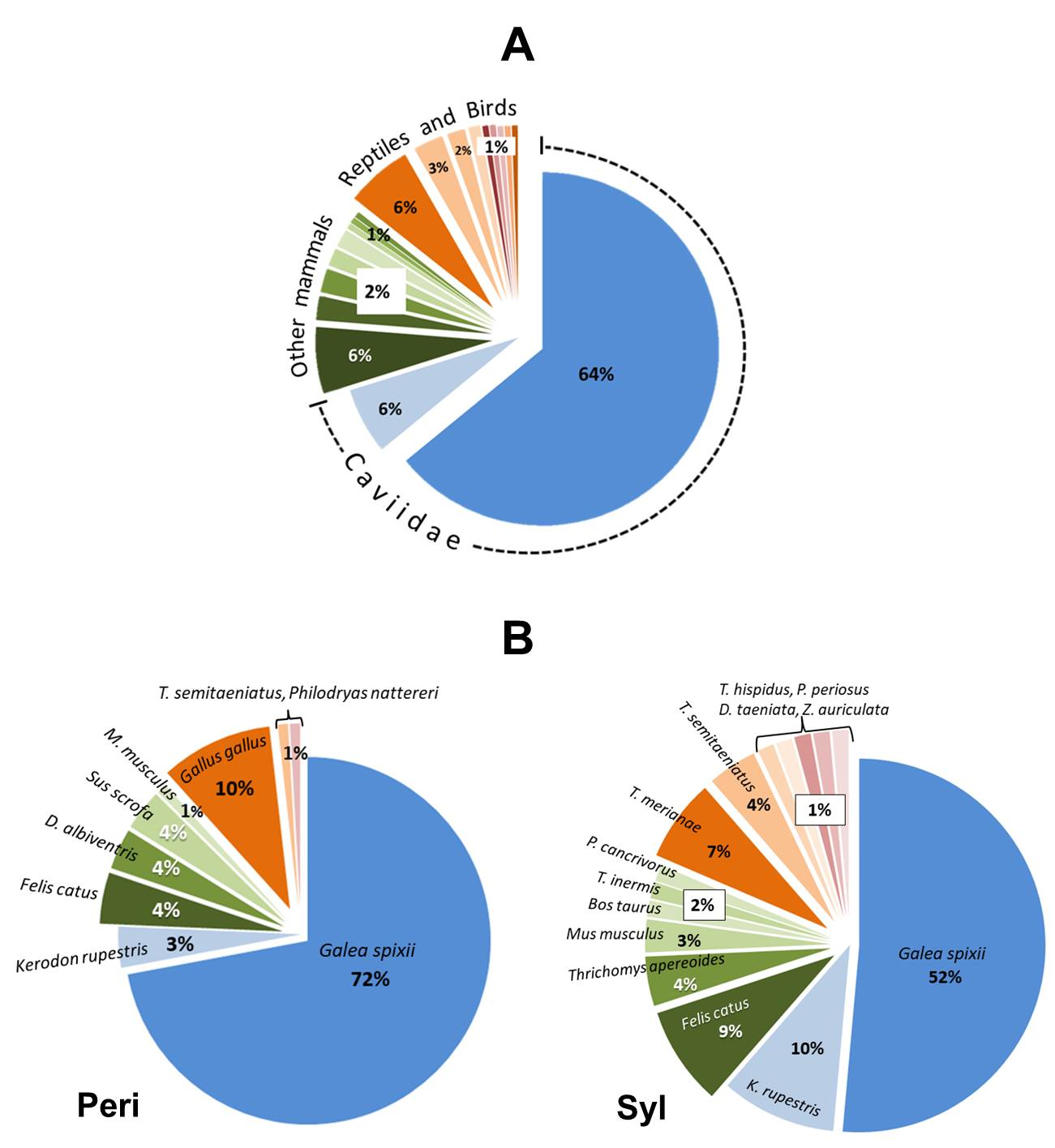

Supplement: S2 File — Overall blood meals (A) and divided by ecotope: Peri = peridomestic and Syl = sylvatic (B). Detailed information from each insect is in S1 File. Rodents of Caviidae Family are in bluish, other mammals are in greenish and reptiles with birds are in hot colors (orange to pinkish). (TIF) [file pntd.0008735.s002.tif]
